# The clinical significance of glutathione peroxidase 2 in glioblastoma multiforme

**DOI:** 10.1515/tnsci-2021-0005

**Published:** 2021-01-20

**Authors:** Bangming Guo, Wenjuan Liao, Shusheng Wang

**Affiliations:** Department of Neurosurgery, The First Affiliated Hospital of Gannan Medical University, Ganzhou, Jiangxi, China; Department of Pediatrics, The First Affiliated Hospital of Gannan Medical University, Ganzhou, Jiangxi, China; Emergency Department, The First Affiliated Hospital of Gannan Medical University, Ganzhou, Jiangxi, China

**Keywords:** prognostic, glioblastoma, GPX2, expression, database

## Abstract

**Background:**

Glioblastoma multiforme (GBM) is the leading cause of death among adult brain cancer patients. Glutathione peroxidase 2 (GPX2), as a factor in oxidative stress, plays an important role in carcinogenesis. However, its role in GBM has not been well established. The study aimed to investigate the clinical significance of GPX2 with GBM prognosis.

**Methods:**

Data of GBM and healthy individuals were retrospectively collected from oncomine, cancer cell line encyclopedia (CCLE), gene expression profiling interactive analysis (GEPIA), UALCAN, and Human Protein Atlas. GPX2 mRNA expression was first assessed across various cancer types in oncomine and cancer cell lines from CCLE. The mRNA expression of GPX2 was compared between normal and GBM tissues using GEPIA (normal = 207; GBM = 163) and UALCAN (normal = 5; GBM = 156). The GPX2 methylation was analyzed using data from UALCAN (normal = 2; GBM = 140). The prognostic value of GPX2 in GBM was explored in GEPIA and UALCAN using Kaplan–Meier method. STRING database was used to construct protein–protein interaction (PPI) network and Kyoto Encyclopedia of Genes and Genomes (KEGG) pathway. Statistical significance was set as <0.05.

**Results:**

The current study revealed no significant differences in GPX2 expression between normal and GBM from GEPIA data (*P* > 0.05) and UALCAN (*P* = 0.257). Patients with higher GPX2 intended to have a poorer prognosis (*P* = 0.0089). The KEGG pathways found that chemokine-signaling pathway were the more preferred.

**Conclusions:**

The findings demonstrated that GPX2 might be a potential diagnosis and prognostic indicator for GBM. Chemokine-signaling pathway may be involved in GPX2 function.

## 1 Introduction

Glioblastoma multiforme (GBM) is the most common form of highly malignant brain tumor with frequent genetic and epigenetic alterations [1,2]. This disease is a leading cause of brain tumors in adults, with a median GBM survival time of less than 16 months, indicating a relatively poor prognosis [3,4]. Numerous genes have been validated to participate in the pathogenesis of GBM, and they serve as markers for differentiation and prognosis of GBM [3,4]. For example, overexpression of epidermal growth factor receptor (EGFR) is found in more than 30% of patients with GBM [5]. GBM tumor cells with EGFR have higher ability of migration and infiltration [5]. And patients with EGFR have a relatively reduced response to therapies, with shorter duration of survival [6]. However, the pathogenesis of GBM remains unclear. Potential contributing genes are encouraged to be explored for this disease.

Oxidative stress plays an important role in the formation and development of malignant tumors [7]. Reactive oxygen species (ROS) are generated by aerobic cells in physiological conditions but are produced in large quantities during external and internal insults, leading to disturbance in DNA, protein, and plasma/organelle membrane [8]. Cells maintain a balance of redox biology by modulating ROS production and elimination. Studies have indicated huge production of ROS and disruption of antioxidant system in cancer cells, because of hypoxia and metabolic alterations. In response to excessive ROS and deficient antioxidant system, tumor suppressors such as p53, phosphatase and tensin homolog (PTEN), and other oncogenes are activated to promote cellular proliferation, transformation, and metastasis [9]. Glutathione (GSH) is the most abundant antioxidative agent in living organisms and is crucial in the removal and detoxification of carcinogens [10]. It is believed that targeting GSH represents a potential treatment option to render cancer cells more sensitive to the standard therapies [8].

Glutathione peroxidase (GPX) includes a family of multiple isozymes that catalyze the reduction of organic hydroperoxides by using reduced GSH as an electron donor [11]. The GPX family has eight isoforms in mammals: GPX1-8. Among them, GPX2 is a gastrointestinal tract-enriched selenium-containing and GSH-dependent enzyme and belongs to the antioxidant enzyme GPX family [11]. GPX2 could reduce the hydroperoxides and exert an antioxidant defense [12,13]. In recent years, the role of GPX2 in cancers has been elucidated. It has been proved that GPX2 overexpression participates in the initiation, development, and metastasis of colorectal, hepatocellular, and bladder cancers [12,14,15]. Moreover, a study by Lei et al. has suggested the role of GPX2 in forming a comprehensive multigene prognosis model for GBM. But the specific expression atlas and prognostic value of GPX2 have not been well established in the disease. Here this study explores the association of GPX2 with GBM.

## 2 Methods

### 2.1 Oncomine

ONCOMINE (https://www.oncomine.org) is an online cancer microarray database designed for translational bioinformatics cancer research. The mRNA expression of GPX2 studied in a series of cancer specimens was compared with normal control tissues, with the following thresholds: the gene rank, *P* value, and fold change were 10%, 0.001, and 2, respectively.

### 2.2 Cancer cell line encyclopedia (CCLE)

The CCLE (https://portals.broadinstitute.org/ccle) is a public database for genomic data analysis and visualization of more than 1,000 cell lines. GPX2 was searched in the website and the expression of the gene from the available cell lines in cancers was exported.

### 2.3 Gene expression profiling interactive analysis (GEPIA)

GEPIA is an interactive web server for analyzing the RNA sequencing expression data of 9,736 tumors and 8,587 normal samples from The Cancer Genome Atlas (TCGA) and the Genotype-Tissue Expression projects, using a standard processing pipeline [16]. The study investigated the differential expression and prognostic values of GPX2 in the modules in the website (http://gepia.cancer-pku.cn/index.html). In all, 207 normal individuals and 163 GBM patients were detected. The coexpressed genes of GPX2 were extracted with the Pearson correlation coefficient of more than 0.60.

### 2.4 Human protein atlas (HPA)

The HPA database is a Swedish-based program started in 2003, aiming at mapping all the human proteins in cells, tissues, and organs with integrative analysis of antibody-based imaging, mass spectrometry-based proteomics, transcriptomics, and systems biology (https://www.proteinatlas.org/) [17,18]. The protein location of GPX2 of normal and GBM tissues was detected using a tissue microarray-based immunohistochemistry method.

### 2.5 UALCAN

UALCAN is a comprehensive, user-friendly, and interactive web resource for analyzing cancer OMICS data (http://ualcan.path.uab.edu/) [19]. It provides easy access to cancer OMICS data (TCGA and MET500), allows users to identify biomarkers to perform in silico validation of potential genes, and evaluates patient survival information, gene methylation, and gene coexpression correlation analysis. GPX2 was searched in UALCAN. The mRNA expression (normal, *n* = 5; GBM, *n* = 156) and gene methylation (normal, *n* = 2; GBM, *n* = 140) were analyzed.

### 2.6 STRING

STRING (https://string-db.org/) is an online database which collects and integrates direct and indirect protein–protein interaction (PPI) in many organisms [13]. The PPI network was constructed and the Kyoto Encyclopedia of Genes and Genomes (KEGG) pathway of GPX2 and its coexpressed genes.


**Ethical approval**: The conducted research is not related to either human or animal use.

### 2.7 Statistical analysis

SPSS 23.0 version software (IBM Analytics) was applied in this study. Student *t* test was used to analyze the differential expression of GPX2 between normal and patients. Correlations between GPX2 and the associated genes were evaluated using Pearson’s correlation analysis. The survival results were displayed with hazard ratios (HRs). A *P* value of less than 0.05 was considered to be statistically significant.

## 3 Results

### 3.1 The expression of GPX2 in GBM

**Figure 1:**
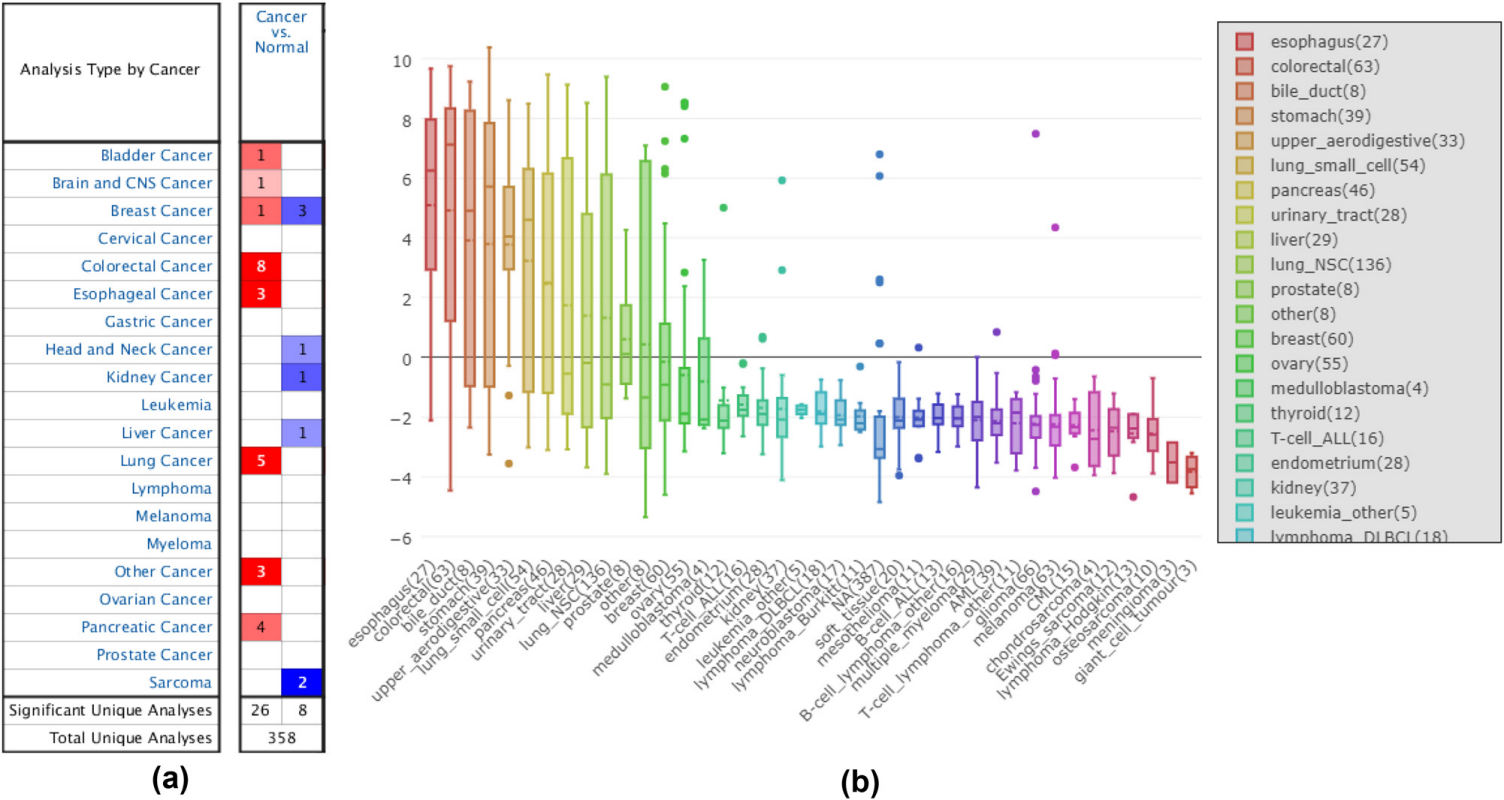
(a) mRNA expression of GPX2 in human cancers. The number in the colored cell means the number of analyses meeting the thresholds. The color depth was determined by the gene rank. The more intense red or blue color indicates an overexpressed or underexpressed gene. (b) The mRNA expression pattern of GPX2 in GBM cell lines from the CCLE database. GPX2, glutathione peroxidase 2; GBM, glioblastoma multiforme; CCLE, cancer cell line encyclopedia.

The study first assessed the differences in mRNA expression of GPX2 in a series of human cancers and normal tissues using the Oncomine database (Figure 1a). It was indicated that GPX2 was moderately elevated in cancers of brain and CNS, compared with that in normal tissues (*P* < 0.05). The project then detected the expression profiles of GPX2 in various cancer cell lines in CCLE (Figure 1b). However, a relatively low expression was seen in the GBM cell lines.

**Figure 2:**
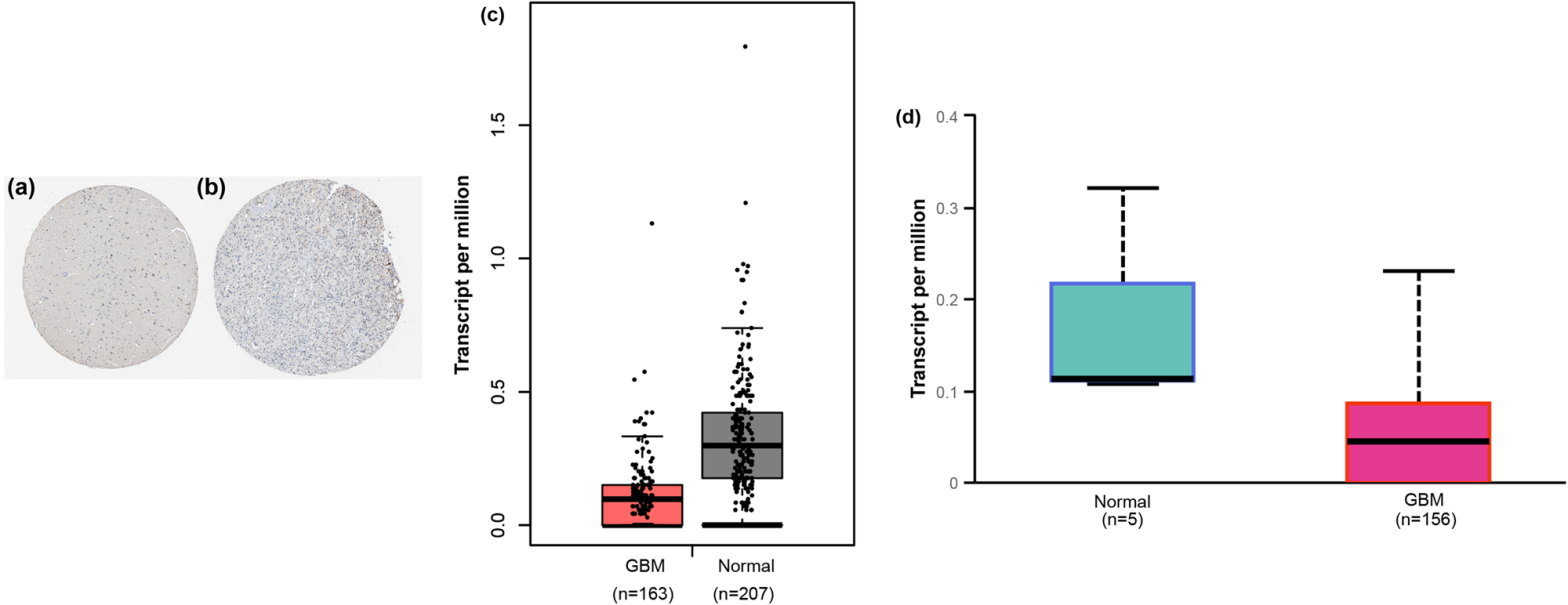
The expression of GPX2 in normal and GBM tissues. Representative immunohistochemical images of GPX2 in normal (a, patient ID: 2521) and GBM tissues (b, patient ID: 2750) were from the HPA database. The scale bars were 25 μm. Photomicrographs were taken at 100× magnification. The GPX2 mRNA expression in GEPIA (c) and CCLE (d). GPX2, glutathione peroxidase 2; GBM, glioblastoma multiforme; HPA, Human Protein Atlas; GEPIA, the gene expression profiling interactive analysis; CCLE, cancer cell line encyclopedia.

The immunohistochemistry images of normal and tumor tissues were from the HPA database and shown in Figure 2. GPX2 was not detected in epithelial cells, glia, and neuropil. A moderate intensity of the protein was seen in the cytoplasm and membrane of neurons (Figure 2a). And a weak staining in tumor cells was seen in Figure 2b. Quantification analysis of GPX2 mRNA level between normal and GBM tissues was done in GEPIA (normal, *n* = 207; GBM, *n* = 163; Figure 2c) and UALCAN (normal, *n* = 5; GBM, *n* = 156; Figure 2d). Although it seemed that a relatively lower mRNA level was seen in both databases, no significances were found (GEPIA, *P* > 0.05; UALCAN, *P* = 0.257).

### 3.2 The methylation of GPX2 in GBM

**Figure 3:**
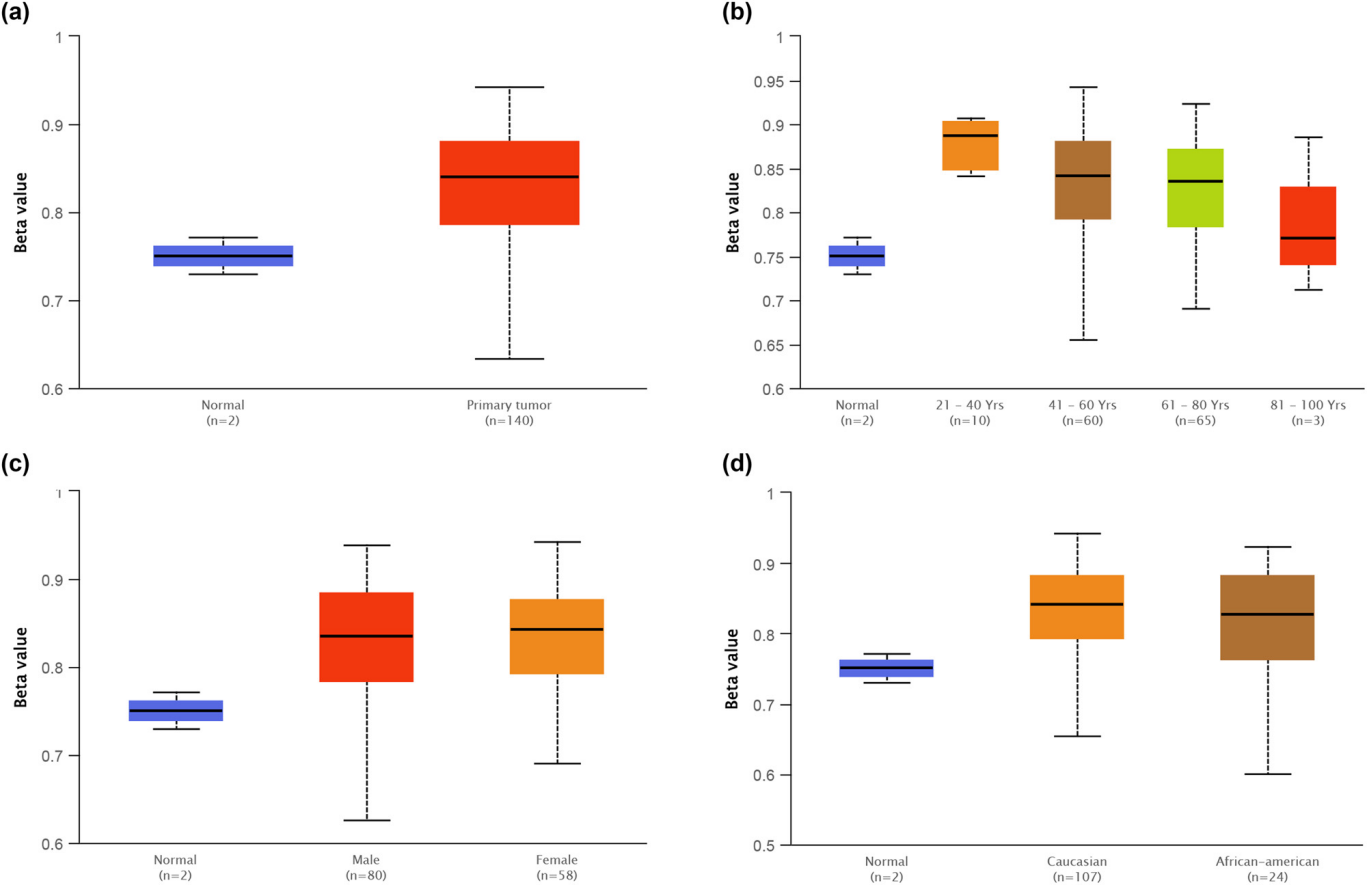
The assessment of GPX2 methylation in GBM. (a) GPX2 methylation between normal and GBM tissues. (b) GPX2 methylation based on age. (c) GPX2 methylation based on gender. (d) GPX2 methylation based on race. The beta value indicates level of DNA methylation ranging from 0 (unmethylated) to 1 (fully methylated). Different beta value cutoff has been considered to indicate hypermethylation (0.7–0.5) or hypomethylation (0.3–0.25). GPX2, glutathione peroxidase 2; GBM, glioblastoma multiforme.

The methylation level of GPX2 in GBM was further analyzed using UALCAN database (normal, *n* = 2; GBM, *n* = 140). No significant differences were indicated with respect to the methylation level of GPX2 in GBM compared with the normal tissues (Figure 3a; *P* = 0.165). Further analysis of methylation conditions based on age (normal vs 21–40 years, *P* = 0.241; normal vs 41–60 years, *P* = 0.143; normal vs 61–80 years, *P* = 0.181; normal vs 81–100 years, *P* = 0.609), gender (normal vs male, *P* = 0.220; normal vs female, *P* = 0.104; male vs female, *P* = 0.525), and race (normal vs caucasian, *P* = 0.166; normal vs African American, *P* = 0.217; Caucasian vs African American, *P* = 0.830) did not reveal any significance (Figure 3b–d).

### 3.3 The prognostic value of GPX2 for GBM

**Figure 4:**
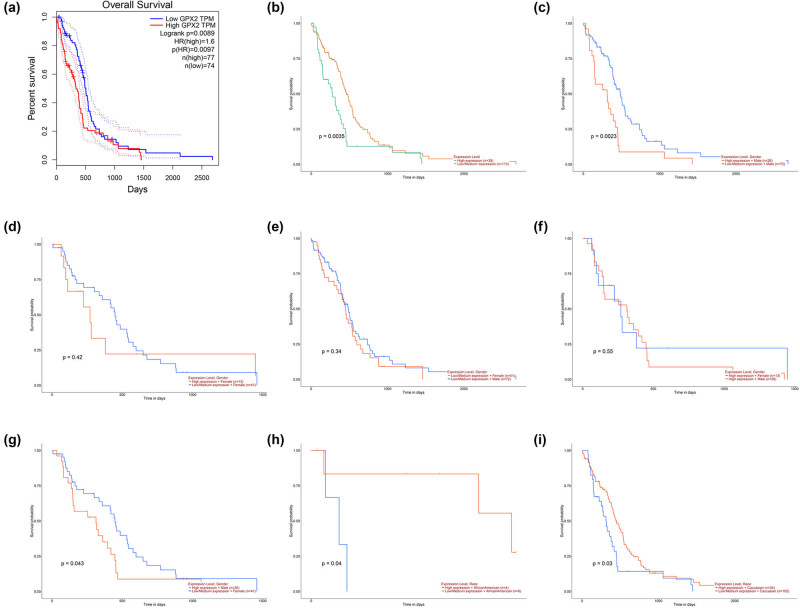
The prognostic values of GPX2 in GBM according to the GEPIA (a) and UALCAN (b). Subgroup survival analysis is done based on gender (c–g) and race (h, i). GPX2, glutathione peroxidase 2; GBM, glioblastoma multiforme; GEPIA, the gene expression profiling interactive analysis; CCLE, cancer cell line encyclopedia.

Next the study investigated the prognostic values of GPX2 for GBM patients using GEPIA and UALCAN databases (Figure 4). It showed that higher GPX2 expression was associated with a shorter overall survival time in all GBM patients (GEPIA, *P* = 0.0089; UALCAN, *P* = 0.0035; Figure 4a and b). Furthermore, subgroup analysis was done based on GPX2 expression level, gender, and race. For male patients, the increasing trend of GPX2 with poor survival was maintained (*P* = 0.0023) but not in the female patients (*P* = 0.42). When, respectively, assessing the effects of gender on GBM with high and low/medium GPX2 expression, no significance was detected (*P* > 0.05). Among Caucasian patients, high GPX2 level was associated with a poor survival rate (*P* = 0.03). However, low GPX2 was correlated with reduced survival rate in African American patients (*P* = 0.04).

### 3.4 Analysis of GPX2-associated coexpressed genes

**Figure 5:**
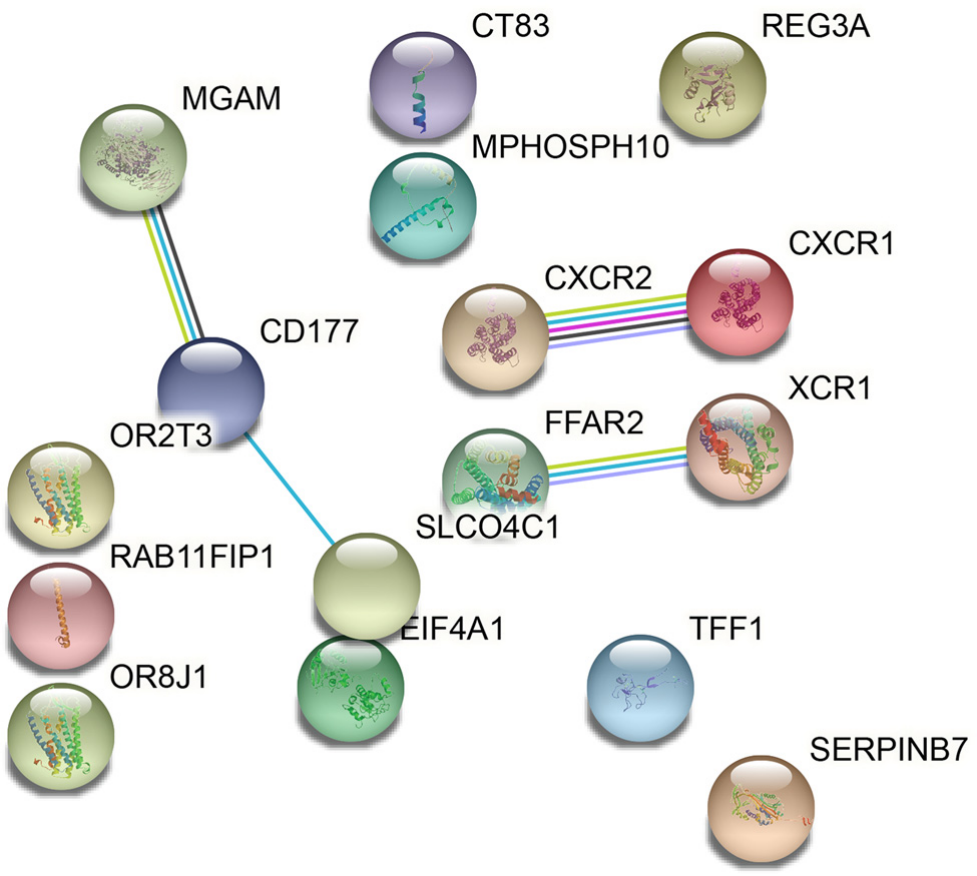
The PPI network of GPX2 and the genes with similar expression pattern. PPI, protein–protein interaction; GPX2, glutathione peroxidase 2.

The coexpressed genes with GPX2 were identified using GEPIA database. First, a total of 200 genes were identified. The cutoff correlation coefficient at 0.60 included 36 genes with similar expression patterns (Table 1). PPI network was established to investigate their association using STRING database (Figure 4). Furthermore, the study analyzed the KEGG pathways of these genes and found that chemokine-signaling pathway (CXCR1, CXCR2, and XCR1) was the more preferred pathway (Table 2; Figure 5).

**Table 1:** The genes with similar expression pattern with GPX2

Gene symbol	Gene ID	PCC
PRSS3P3	ENSG00000277083.1	0.7
RP11-325O24.6	ENSG00000281248.1	0.7
CT83	ENSG00000204019.4	0.7
RP11-666A8.11	ENSG00000267084.1	0.7
LINC01360	ENSG00000233973.5	0.7
U3	ENSG00000281710.1	0.7
AC055736.3	ENSG00000281194.1	0.7
SNX19P1	ENSG00000228184.1	0.7
RP11-799D4.2	ENSG00000266981.1	0.7
RPS4XP12	ENSG00000244400.2	0.7
RP11-305F5.2	ENSG00000243016.1	0.7
RN7SL653P	ENSG00000239794.3	0.7
RP11-353N14.3	ENSG00000262343.1	0.7
AC093698.4	ENSG00000224529.1	0.7
OR8J1	ENSG00000172487.3	0.7
OR4A7P	ENSG00000255349.1	0.7
RP4-591B8.3	ENSG00000271419.1	0.7
OR2T3	ENSG00000196539.3	0.7
PRPF19P1	ENSG00000264685.1	0.7
AC068044.1	ENSG00000223691.1	0.68
SLCO4C1	ENSG00000173930.8	0.68
RP4-568C11.4	ENSG00000274173.1	0.68
SNORA48	ENSG00000252774.1	0.67
CTD-2066L21.2	ENSG00000251281.1	0.66
FFAR2	ENSG00000126262.4	0.65
CXCR2	ENSG00000180871.7	0.64
XCR1	ENSG00000173578.7	0.63
RP11-34P13.9	ENSG00000241599.1	0.63
CXCR1	ENSG00000163464.7	0.62
REG3A	ENSG00000172016.15	0.61
RAB11FIP1	ENSG00000156675.15	0.61
TFF1	ENSG00000160182.2	0.61
CD177	ENSG00000204936.9	0.61
SERPINB7	ENSG00000166396.12	0.6
MGAM	ENSG00000257335.8	0.6

PCC, Pearson correlation coefficient; GPX2, glutathione peroxidase 2.

**Table 2:** KEGG pathways associated with GPX2 coexpressed genes in GBM

Term	Description	Gene count	False discovery rate	Matching proteins in network
hsa04062	Chemokine-signaling pathway	3	0.0086	CXCR1, CXCR2, XCR1
hsa04060	Cytokine–cytokine receptor interaction	3	0.0098	CXCR1, CXCR2, XCR1
hsa04144	Endocytosis	3	0.0098	CXCR1, CXCR2, RAB11FIP1
hsa05120	Epithelial cell signaling in *Helicobacter pylori* infection	2	0.0098	CXCR1, CXCR2
hsa04072	Phospholipase D-signaling pathway	2	0.024	CXCR1, CXCR2

KEGG, Kyoto encyclopedia of genes and genomes; GPX2, glutathione peroxidase 2; GBM, glioblastoma multiforme.

## 4 Discussion

This study summarized data from public databases and discovered the clinical performances of GPX2 in GBM patients and their prognosis. It also indicated that GPX2 methylation was lower in GBM patients. In addition, the study analyzed the GPX2-coexpressed genes and established the network’s analysis of these genes.

GPX is composed of a series of isozymes (GPX1-8) that catalyze the reduction in H_2_O_2_ or organic hydroperoxides to water or corresponding alcohols [11,20]. Among them, GPX2 is a gastrointestinal tract-enriched selenium-containing and GSH-dependent antioxidant enzyme [11]. Recently, it attracts increasing attention for its involvement in cancer formation and progression, as an ROS scavenger. The protein has been validated in the pathogenesis of many types of cancers, such as rectal cancer and bladder cancer [14,21]. This study confirmed the expression of GPX2 in GBM tissues. It seems that a relatively lower expression of the molecule is detected in GBM compared to normal samples, from the results in GEPIA and HPA databases. However, no significance was revealed. It is quite different from the expression analysis that upregulation of GPX2 level is found in epithelium-derived carcinomas and involved in carcinogen-induced tumor initiation, tumor growth, and metastasis [13,14,21]. This implies the variety of GPX2 features in different types of cancers.

In this study, the survival analysis was based on GPX2 expression level in GBM patients. Significant associations of higher GPX2 expression were detected with poorer prognosis. This is consistent with the previous studies, emphasizing the participation of GPX2 in GBM. A study by Lei et al. had pointed the prognostic value of GPX2 for GBM patients. They screened GPX2 as a favorable gene candidate, and the expression level of GPX2 was negatively related to the overall survival status. But no subgroup analysis was done in that study. We here found the trends of prognostic analysis get fluctuated based on gender. This may imply the role of gender on the effects of GPX2. Studies have shown that sex hormones and sex chromosomes can have intrinsic control of cancer-initiating cell populations, tumor environment, and cancer development determinants [22]. The differences have also been observed in oxidative stress and metabolism [23,24]. It may be speculated that sex hormones may participate in the GPX2’s tumor-related functions. Further exploration is required to understand how the hormones will influence GPX2 effects. Furthermore, GPX2 demonstrates inverse relationship with the survival results in African American patients that reduced the level of the protein associated with poorer survival time, compared to the overall and Caucasian patients. However, the sample size was somewhat small. More samples should be included in the future.

Additionally, the study displayed the coexpressed genes with GPX2 in the PPI network. KEGG analysis of the coexpressed genes of GPX2 showed that the main enriched biological pathway was chemokine-signaling pathway. Chemokines are chemotactic cytokines synthesized by various types of cells, including monocytes, macrophages, T lymphocytes, and neutrophils. Chemokine receptors belong to G-protein-coupled receptor (GPCR) family. Several studies have pointed out the association of GPX2 with GPCR. For example, Lennicke et al. [25] found that the depletion of GPX2 downregulates the expression of leucine-rich repeat-containing GPCR 5 (Lgr5). Following receptor activation, the alpha- and beta-gamma subunits of G protein dissociate to activate the diverse downstream pathways in cellular polarization and actin reorganization, which are important functions for tumor formation and development [26]. Increasing evidences have suggested that chemokines and their receptors play essential roles in the cellular interactions associated with inflammation and carcinogenesis [27]. CCL2/CCR2 axis plays a fundamental role in GBM and the migration of monocytes from the bloodstream through the vascular endothelium [28]. The cross-talk between Notch1 signaling and CXCL12/CXCR4 system contributes to the progression and recurrence of GBM by promoting the self-renewal and invasion of glioma stem cells [29]. However, further studies on GPX2 in GBM could be done regarding these targets.

## 5 Conclusions

This study offers a new idea for the possible mechanism of human GBM. The overall results indicate that GPX2 is a candidate proto-oncogene for GBM. GPX2 might be a potential therapeutic target for GBM patients.
